# Effective coordination numbers from EXAFS: general approaches for lanthanide and actinide dioxides

**DOI:** 10.1107/S160057752101300X

**Published:** 2022-01-27

**Authors:** Anna Romanchuk, Alexander Trigub, Tatiana Plakhova, Anastasiia Kuzenkova, Roman Svetogorov, Kristina Kvashnina, Stepan Kalmykov

**Affiliations:** a Department of Chemistry, Lomonosov Moscow State University, Leninskie Gory 1, Bld. 3, Moscow 119991, Russian Federation; b National Research Centre ‘Kurchatov Institute’, Pl. Kurchatova 1, Moscow 123182, Russian Federation; c The Rossendorf Beamline at ESRF – The European Synchrotron, CS40220, 38043 Grenoble Cedex 9, France; d Institute of Resource Ecology, Helmholtz-Zentrum Dresden-Rossendorf (HZDR), PO Box 510119, 01314 Dresden, Germany

**Keywords:** extended X-ray absorption fine structure (EXAFS), actinide, plutonium, cerium, nanoparticles, coordination number

## Abstract

Extended X-ray absorption fine structure (EXAFS) is a comprehensive and usable method for characterizing the structures of various materials, including radioactive and nuclear materials. Unceasing discussions about the interpretation of EXAFS results for actinide nanoparticles (NPs) or colloids were still present during the last decade. In this study, new experimental data for PuO_2_ and CeO_2_ NPs with different average sizes were compared with published data on AnO_2_ NPs that highlight the best fit and interpretation of the structural data. In terms of the structure, PuO_2_, CeO_2_, ThO_2_, and UO_2_ NPs exhibit similar behaviors. Only ThO_2_ NPs have a more disordered and even partly amorphous structure, which results in EXAFS characteristics. The proposed new core-shell model for NPs with calculated effective coordination number perfectly fits the results of the variations in a metal–metal shell with a decrease in NP size.

## Abstract 2

New experimental EXAFS results for PuO_2_ and CeO_2_ nanoparticles in the size range of 2 nm were compared with published data for other lanthanide and actinide dioxides. A conceptual core-shell model with a calculated effective coordination number is proposed to fit the changes in EXAFS.

## 1. Introduction

Plutonium dioxide (PuO_2_) is a crucial component of modern atomic energy (Clark *et al.*, 2005 ▸, 2019 ▸; McFarlane, 2004 ▸). A possible strategy for the nuclear power cycle is to use mixed-oxide fuels that contain 3–5% PuO_2_ (Carbajo *et al.*, 2001 ▸). In contrast, PuO_2_ in particulate and colloidal forms is important in the context of radioecology and environmental safety. Colloidal transport of plutonium in the environment was found to be the predominant mechanism of subsurface migration (Kersting *et al.*, 1999 ▸; Novikov *et al.*, 2006 ▸). These findings make it necessary to conduct careful studies of both pseudo- and intrinsic plutonium-containing colloidal particles.

However, despite its practical importance, PuO_2_ is even more intriguing from a fundamental perspective. Indeed, considering the periodic system, it is not easy to find cations that can be present as Me^4+^ (where Me indicates a metal) in aqueous solutions. This list mainly includes Th^4+^, U^4+^, Np^4+^, Pu^4+^, and one lanthanide Ce^4+^. All tetravalent lanthanides and actinides, such as Th, U, Np, Pu, and Ce, have a high tendency to form MeO_2_ precipitates in the form of nanoparticles (NPs) (Powell *et al.*, 2011 ▸; Romanchuk *et al.*, 2018 ▸; Gerber *et al.*, 2020 ▸), or sometimes referred to as polymers or eigen/intrinsic colloids (Silver, 2001 ▸; Rai & Swanson, 1981 ▸; Costanzo *et al.*, 1973 ▸; Triay *et al.*, 1991 ▸; Thiyagarajan *et al.*, 1990 ▸). Meanwhile, plutonium stands out in this series because of its complicated chemistry with redox reactions and high radiotoxicity.

Extended X-ray absorption fine structure (EXAFS) is a powerful method for characterizing the local structure of nanostructured materials (Kuzmin & Chaboy, 2014 ▸; Rehr & Albers, 2000 ▸; Lee *et al.*, 1981 ▸). This method is element-selective, non-destructive, and relatively moderate for the sample during measurements (no vacuum, heating, and ionization), making it one of the most popular and widely used techniques for characterizing radioactive materials.

Although EXAFS has been widely used for the characterization of PuO_2_, compared with other methods, the interpretation of the results is still debatable. Conradson *et al.* (2004 ▸) distinguished up to eight individual components in the first coordination shell of plutonium dioxide, which corresponds to the interaction of plutonium atoms with neighboring oxygen atoms. The authors proposed a chemical formula for the colloids as PuO_2+*x*–*y*_(OH)_2*y*_·*z*H_2_O, where *x* indicates the Pu(V) species due to the presence of the Pu(V)–O, plutonoyl, component at ∼1.9 Å. However, in the study by Rothe *et al.* (2004 ▸), this short distance was attributed to multi-electron excitation and thus excluded from consideration. They isolated two components from EXAFS, the first with a Pu–O distance of 2.20–2.24 Å, and the second with a Pu–O distance of 2.38–2.42 Å. The shorter distance was attributed to the interaction of the plutonium atom with the hydroxo-group or with the oxygen atom of the water molecule. In contrast, Hudry *et al.* (2014 ▸) isolated only one component, Pu–O, in the EXAFS spectrum of PuO_2_ nanocrystals with a distance of 2.31 Å, which is slightly less than that of bulk PuO_2_. A slight decrease in the coordination number (CN) in the first and second coordination shells was observed. Dalodière *et al.* (2017 ▸), in their study of PuO_2_ NPs obtained by both hydrolytic and sonolitic methods, reported a split in the first coordination shell of plutonium and identified three different interatomic interactions: short (1.93–2.23 Å), medium (2.23–2.63 Å), and long (2.63–3.13 Å) ranges. In this case, the short component corresponds to μ_1_-Pu–OH or μ_3_-Pu–O, the medium component corresponds to μ_4_-O from the PuO_2_ ideal structure, and the long component corresponds to the surface-adsorbed H_2_O molecules. In the continuation of this study, Bonato *et al.* (2020 ▸) suggested that the splitting is due to the disordered crystal structure of the NPs. Recently, the interpretation of EXAFS spectra was revised by Micheau *et al.* (2020 ▸). The authors used a single Pu–O scattering path to fit a Fourier-filtered oxygen shell and determined the corresponding Debye–Waller factor (DWF) as the only floating parameter. In the paper by Gerber *et al.* (2020 ▸), using different approaches including Landweber iteration and Monte Carlo simulation proved the absence of legitimate reasons to split the Pu–O shell in PuO_2_ NPs.

This study aims to compare EXAFS data for tetravalent CeO_2_, ThO_2,_ UO_2_, and PuO_2_ NPs of various sizes and compare several approaches for fitting the EXAFS spectra.

## 2. Experimental

### 2.1. Synthesis and characterization of CeO_2_ and PuO_2_ NPs of various sizes

Cerium dioxide NPs were synthesized via rapid chemical precipitation. In this synthesis approach, both the type and concentration of the starting salt affect the particle size. Cerium (IV) ammonium nitrate, (NH_4_)_2_Ce(NO_3_)_6_, and cerium (III) nitrate hexahydrate, Ce(NO_3_)_3_·6H_2_O, were used to prepare the initial cerium solutions. Concentrations of the salts varied from 0.01 to 0.8 *M*. Aqueous solutions of the cerium salts were added to 3 *M* aqueous solution in molar excess under constant stirring, resulting in the formation of yellow suspensions. The precipitates were separated by centrifugation and washed three times with Milli-Q water to remove any impurities. For further measurements, the samples were air-dried for 24 h at 40°C. A sample synthesized from 0.1 *M* (NH_4_)_2_Ce(NO_3_)_6_ was additionally annealed for 12 h at 400°C in a muffle furnace.

Plutonium dioxide was formed as a result of the long storage (375 days) of Pu(VI) solution at a total Pu concentration of 10^−4^ *M* at pH ∼8 and 12.

Synchrotron-based X-ray diffraction (XRD), performed at the XSA beamline (Svetogorov *et al.*, 2020 ▸) of the Kurchatov Synchrotron Radiation Source (Moscow, Russia) using a Rayonix SX165 detector, was employed to characterize the inorganic matrix of the bottom sediments. Diffraction patterns were obtained using monochromatic radiation with a wavelength of λ = 0.8 Å focused on a spot of 400 µm of a sample held in a polymer capillary in the case of Pu-containing substances and a cryoloop in the case of CeO_2_. Two-dimensional diffraction patterns were further transformed using *Dionis* software to reveal the dependence of the intensity on the scattering angle.

The average particle size of the CeO_2_ NPs was calculated from the XRD data using different procedures. It was calculated from the broadening of the diffraction lines using both the Scherrer equation and Williamson–Hall approach. The full width at half-maximum (FWHM) parameter was estimated from the diffraction peaks fitted by the pseudo-Voigt function. Instrumental broadening was calculated using the Caglioti formula (Caglioti *et al.*, 1958 ▸) and considered when calculating the particle size by direct subtraction from the FWHM values. Determination of the unit-cell parameters and calculation of the values of the crystallite size and microstress influence were performed using Rietveld refinement in the *Jana2006* software (Petříček *et al.*, 2014 ▸) (see example in Fig. S1C of the supporting information). Instrumental broadening was determined using the LaB6 certified crystallographic standard (NIST SRM 660a). A comparison of the CeO_2_ NP sizes determined by different approaches is summarized in Table S1 of the supporting information. The size of the PuO_2_ crystallites was estimated from the broadening of the first four diffraction peaks [(111), (200), (220), and (311)] using the Scherrer equation. In the PuO_2_ XRD data, a substantial contribution of the background diffraction scattering from the capillary was observed. Therefore, adequate subtraction of the background during the data procedure is unattainable.

### 2.2. EXAFS measurement

XAFS spectra were collected at the Structural Materials Science beamline (Chernyshov *et al.*, 2009 ▸) of Kurchatov Synchrotron Radiation Source (Moscow, Russia). A storage ring with electron beam energy of 2.5 GeV and current in the range 80–100 mA was used. Pu *L*_3_-edge XAFS was measured using an X-ray beam monochromated with a Si(220) channel-cut monochromator, which provided an energy resolution of Δ*E*/*E* ≃ 2 × 10^−4^. The damping of higher-energy harmonics was achieved by monochromator geometry distortion. The XAFS spectrum of the Zr foil was used for energy calibration. Ce *L*-edges have a very short energy range (440 eV for the *L*_3_-edge); therefore, only a few parameters could be extracted from the EXAFS spectra measured at Ce *L*-edges. The Ce *L*_3_-edge spectrum also contains the contribution of the multi-electron effect, which should be considered during data treatment. Therefore, Ce *K*-edge XAFS measurements were inspired by the possibility of measuring the EXAFS spectra over a wide *k*-range. Ce *K*-edge XAFS was measured using a Si(333) channel-cut monochromator, and the Si(111) reflection was annihilated by an Al filter with a thickness of 5 mm. All the experimental data were collected in transmission mode using ionization chambers filled with an appropriate mixture of Ar/N_2_ for the Pu *L*_3_-edge and Xe for the Ce *K*-edge. At every energy point in the XANES region the signal was integrated for 1 s, whereas for the EXAFS region the integration time was set to 1 s at the beginning of the region and increased to 4 s at the end of the region. Samples for Pu *L*_3_ measurements were stored in the polymer capillaries during the measurements, but CeO_2_ powders were pressed into the pallets with appropriate thickness. The beam size for the Pu *L*_3_ X-ray absorption spectrometry (XAS) was selected to be suitable for the homogeneous area of the samples, but not less than 500 µm × 500 µm to obtain the appropriate signal-to-noise ratio. For the Ce *K*-edge XAS experiments, we used a beam with a size of 1 mm × 4 mm. For all samples, at least three spectra were collected and merged using *IFEFFIT* software (Newville, 2001 ▸).

## 3. Results and discussion

To distinguish the influence of the particle size on the spectral characteristics, samples of CeO_2_ and PuO_2_ with different average particle sizes were studied by XRD. In all the cases, a fluorite-type diffraction pattern was observed (Fig. S1) with a different line broadening, from which the average crystallite size was calculated as described in the *Experimental* section[Sec sec2] and Table S1. Consequently, six samples of CeO_2_ were selected with particle sizes ranging from ∼2 to 20 nm (Table 1 ▸). In the case of PuO_2_, the particle size varied from 2.0 to 3.2 nm. Bulk samples of CeO_2_ and PuO_2_ were used for comparison. The XANES spectra for size series look very similar (Fig. S2).

**Figure 1:**
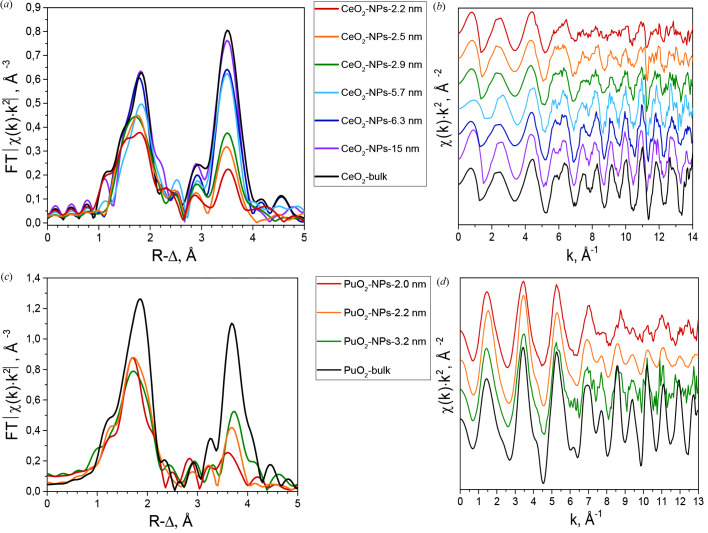
(*a*, *b*) Ce *K*-EXAFS: (*a*) FT magnitude of EXAFS data (*k* = 3–13), (*b*) *k* ^2^-weighted χ(*k*) experimental functions; (*c*, *d*) Pu *L*_3_-EXAFS: (*c*) FT magnitude of EXAFS data (*k* = 3–13), (*d*) *k* ^2^-weighted χ(*k*) experimental functions.

The magnitudes of the Fourier transform (FT) of the weighted experimental EXAFS spectra for the studied CeO_2_ and PuO_2_ NPs are shown in Fig. 1 ▸. In all cases, two main shells are clearly distinguished: Me–O with the maximum approximately *R* − Δ = 1.8 Å and Me–Me with the maximum approximately *R* − Δ = 3.7 Å. Additional peaks or shoulders at 1.15–1.2 Å in the case of PuO_2_ spectra result from atomic contributions or multielectron excitations (Rothe *et al.*, 2004 ▸). Spectral features between 2 and 3 Å could be attributed to the complicated shape of the contributions of heavy elements (metal in the second coordination shell) and multiple-scattering paths (Bocharov *et al.*, 2017 ▸). The contribution of the multiple-scattering paths is visible in the experimental data, but is not essential for modeling the EXAFS spectra of actinide dioxide NPs. With decreasing size, the second coordination shell drastically decreases. The first coordination shell is changed to a decreasing size, but the changes are less definite in this case. The intensity reduction is caused by the decrease in the average CNs and the distortion in the atomic structure of the NP due to the size effect. The explanation and description of both effects can be found in the appropriate sections.

The following approach was used to fit the experimental EXAFS spectra. First, the EXAFS spectra corresponding to the bulk samples were fitted. In this case, the CNs for the two nearest coordination shells were fixed, but the DWFs and interatomic distances to absorbing atoms were varied. The CNs for the Me–O and Me–Me shells were fixed as they should be in the ideal crystal. From this fitting procedure, the DWF for the Me–Me shell was extracted, and they were later used to fit the EXAFS spectra of the NP samples. All spectra were fitted in the *R*-space with *k*-weights of 2 and 3 using symmetric square windows with ‘Hanning sills’.

EXAFS spectra for NPs were fitted with varying radii of Me–O and Me–Me coordination shells, DWF for Me–O coordination shells, and CN for Me–Me shells. Four parameters were optimized by fitting the NP EXAFS spectra. The proposed procedure dramatically decreases the number of variables and obtains stable values of fitted structural parameters, which is essential for further reliable structural data treatment. The amplitude reduction factor, 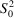 , was defined as 0.9 in the *FEFF* calculation and fixed at that value in the data fits, which is typical for actinides and for *K*-edges of heavy-atom EXAFS spectra fitting (Prieur *et al.*, 2019 ▸). The energy shift parameter (Δ*E*) was treated as a variable for the bulk sample, and the obtained value was used for the NPs.

Similar trends were obtained for the CN of Me–Me when the DWF was varied (see Fig. S4). However, relatively high uncertainties in such determination prevent the understanding of the tiny effects on the structure changes with the decrease in the particle size.

The obtained results are summarized in Table 2 ▸ and Fig. S3.

### 3.1. First oxygen shell (Me–O)

Interpretation of the first Me–O shell in the case of actinide dioxide NPs provokes an intense scientific discussion. A high DWF and non-symmetry indicate extraordinary structural features. As discussed in the *Introduction* section[Sec sec1], some authors proposed to fit this shell by combining several different distances (Conradson *et al.*, 2004 ▸; Dalodière *et al.*, 2017 ▸; Rothe *et al.*, 2004 ▸, 2009 ▸). One of the possible interpretations of the presence of oxidized Pu(V) or Pu(VI) in the structure of PuO_2_ NPs was confidently rejected (Gerber *et al.*, 2020 ▸; Bonato *et al.*, 2020 ▸) and will not be further considered in this study. To avoid contrived conclusions resulting from overfitting, some authors have proposed that the first coordination oxygen shell is not split into different subshells (Gerber *et al.*, 2020 ▸; Bonato *et al.*, 2020 ▸; Micheau *et al.*, 2020 ▸). Here, we followed the same strategy. We avoided the isolation of this Me–O shell as in previous studies (Bonato *et al.*, 2020 ▸; Micheau *et al.*, 2020 ▸), but fixed the Me–O CN and made a variable DWF that is different from Gerber *et al.* (2020 ▸).

**Figure 2:**
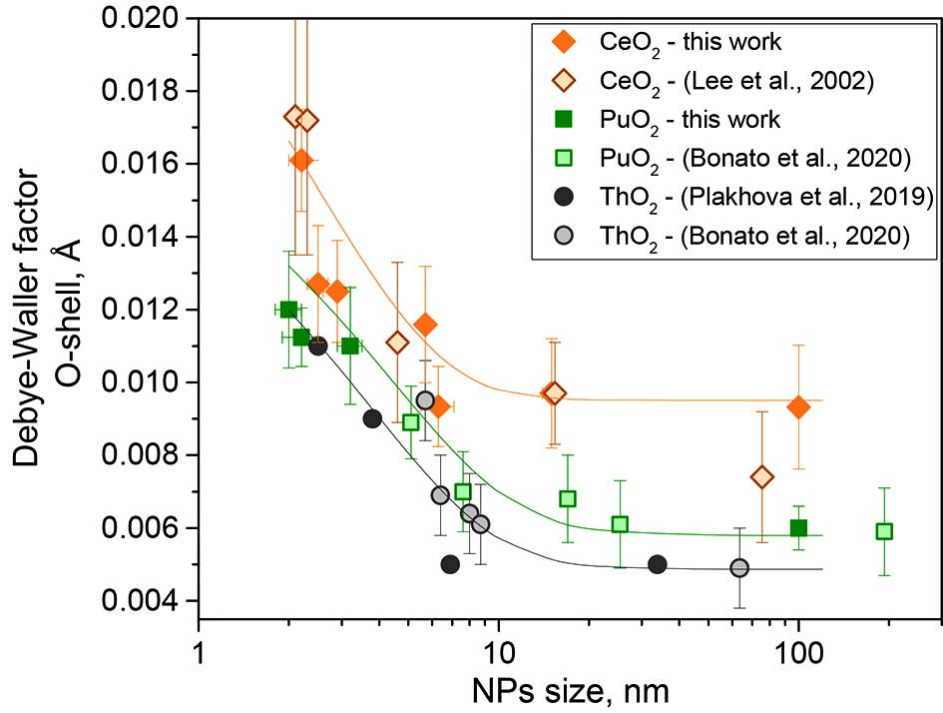
Dependence of the DWF of the first coordination shell (Me–O) with the average NP size from the results of this work and previously published data. ▸

Consequently, we showed in this study that the DWF is essentially increased with decreasing NP size (Table 2 ▸), which is even more pronounced in the case of CeO_2_ NPs. The results of this work were compared with previously published data (Fig. 2 ▸), along with ThO_2_.

Despite the slightly different approaches, the results converge well. All NPs — CeO_2_, PuO_2_, and ThO_2_ — maintained the same trend. With a decrease in the size of the NPs to less than 10 nm, the DWF of the first coordination shell is drastically increased. Such an increase in the DWF correlates with the increasing contribution of the surface atoms (Fig. S5), which indirectly indicates that the disordering effect in the oxygen shell is related to the surface atoms.

Another observation is that DWFs for Me–O shells are generally lower for ThO_2_ NPs than for PuO_2_, and the DWF values for CeO_2_ are consistently higher, even taken from the works of different authors. Additionally, the first shell in the spectra of ThO_2_ NPs (Fig. S6) is much more symmetrical than that in CeO_2_, where the splitting appears to be clear.

### 3.2. Second coordination shell (Me–Me)

**Figure 3:**
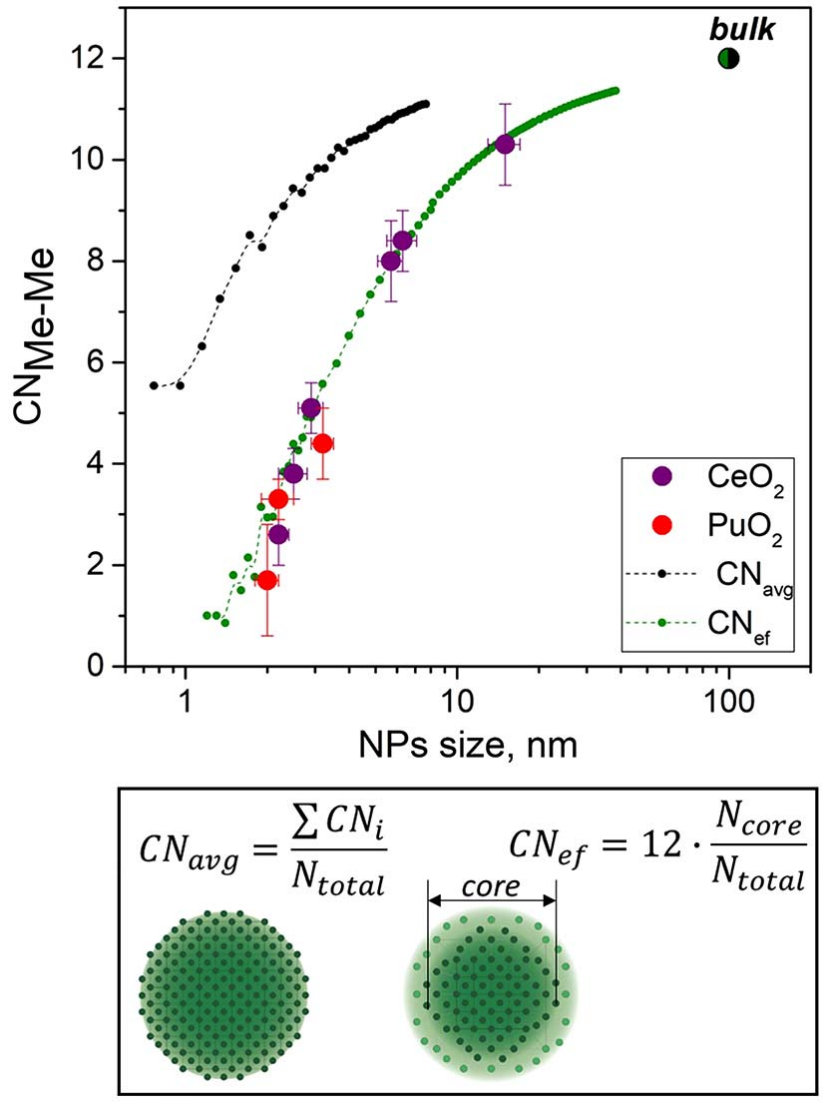
Comparison of the size-dependent change of Me–Me CN in CeO_2_ and PuO_2_ NPs, as determined by EXAFS versus calculated values.

The CNs of the second Me–Me coordination shell obtained from EXAFS spectra fitting were compared for PuO_2_ and CeO_2_ NPs of various sizes (Fig. 3 ▸). As discussed, CN_Me–Me_ decreased with a decrease in the particle size. To explain this fact, experimental data were compared with the calculated values for spherical NPs as a function of particle size (CN_avg_). Upon reduction of the NP size, the number of undercoordinated atoms located on the surface increases relative to those in the bulk, thus leading to a decrease in the average CN (Kuzmin & Chaboy, 2014 ▸). This relatively simple geometric consideration did not converge to the experimental data. A similar difference between the experimental CN and the calculated values was observed for MoS_2_ (Shido & Prins, 1998 ▸) and in one of our previous studies on the size-dependent series of ThO_2_ NPs (Plakhova *et al.*, 2019 ▸).

To address this problem, we propose a structural model of MeO_2_. Presumably, MeO_2_ NPs have a core-shell structure. Metal atoms were suggested to belong to the core of the NP when they contain 12 metal atoms in the second coordination shell; otherwise, metal atoms belong to the shell of the MeO_2_ NP. The idea is that only the core Me atoms contribute to the net Me–Me CN, but all the Me atoms in the MeO_2_ NP contribute to the overall EXAFS signal. Therefore, the effective Me–Me CN (CN_ef_) can be calculated by normalizing the sum of the CNs for core atoms to the total number of Me atoms in the particle. The suggested effective CN can be calculated using the following equation,

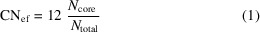

where *N*_core_ denotes the number of Me atoms in the core and *N*_total_ is the total number of Me atoms in the MeO_2_ NP.

This assumption is in excellent agreement with the experimental results for the PuO_2_ and CeO_2_ NPs. Furthermore, this assumption suggests that the EXAFS technique is more sensitive to the highly ordered crystalline core, at least in the case of a second coordination shell.

Using the given assumption, the size of this shell could be estimated as the difference between the radii of the NP and its core part. This calculation provides a value of approximately 0.4 nm, which is close to the cell parameter of the MeO_2_ crystal structure (Table 3 ▸). Recent work (Micheau *et al.*, 2020 ▸) also provided insight into the core-shell structure of PuO_2_ NPs and determined their size using small-angle X-ray scattering (SAXS). In this study, the size of the shell was estimated to be relatively large (approximately 1.0–1.5 nm) and could be interpreted by the presence of a less-ordered shell near the core and a double electric layer near the particles, which is usually accounted for by the SAXS technique.

Notably, the results for PuO_2_ and CeO_2_ converged well with each other. This indicates that cerium dioxide can be considered an appropriate analog for PuO_2_ NPs.

**Figure 4:**
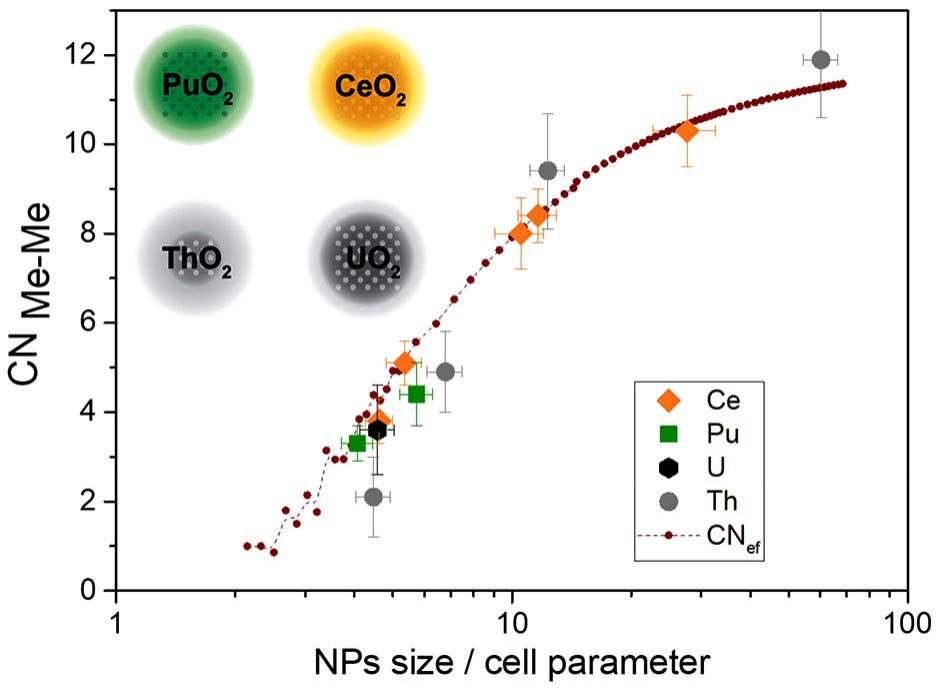
Changes in Me–Me CN with NP size in the series UO_2_–ThO_2_–CeO_2_–PuO_2_. Data for ThO_2_ (Plakhova *et al.*, 2019 ▸) and UO_2_ NPs (Gerber *et al.*, 2021 ▸) were taken from our previously published papers.

A comparison of the results presented here and published previously for ThO_2_ (Plakhova *et al.*, 2019 ▸) and UO_2_ NPs (Gerber *et al.*, 2021 ▸) is shown in Fig. 4 ▸. Because the cell parameters for the studied dioxides were slightly different (Table 3 ▸), the NP size values were divided by the cell parameter of bulk MeO_2_ and used for the *x*-axis (Fig. 4 ▸). The clear trend for all series, that is, CeO_2_–PuO_2_–ThO_2_–UO_2_, remained the same. These results suggest that the proposed core-shell model for CN_ef_ is adequate for all the studied MeO_2_ NPs. Moreover, the results presented here confirm that all NPs have similar structural properties.

In the case of ThO_2_, the CN of smaller NPs (less than 10 nm) was slightly lower than that of the other studied dioxides. Notably, for the Me–O interaction, ThO_2_ NPs have a lower DWF than the other studied NPs (presented above), whereas the Me–Me interaction is less pronounced or more disordered. All these findings suggest that, in ThO_2_ NPs, oxygen atoms are more ordered than more distant atoms, indicating that ThO_2_ has a more amorphous structure than the other metal dioxides studied. In our latest work (Amidani *et al.*, 2021 ▸) on ThO_2_ NPs using the pair distribution function (PDF) method extracted from high-energy X-ray scattering (HEXS) data, we show that for samples containing very small NPs the first Th–O interaction has a higher intensity than normal. This also indicates better ordering of the first oxygen surroundings. The presence of thorium clusters in a mixture with ThO_2_ NPs was proposed to fit the PDF data. Similar effects may be present in cases where an increase in the amorphous thickness of the shell near the ThO_2_ crystalline core occurs. Therefore, it is more evident that ThO_2_ has a more amorphous nature than CeO_2_, PuO_2_, and UO_2_. This conclusion has a good correlation with the weakness of the cation Th^4+^ compared with the other studied cations. This chemical weakness of the Th^4+^ cation also results in other macro properties, such as higher solubility (higher log*K*_sp_) compared with PuO_2_ and CeO_2_ (Table 3 ▸).

## 4. Conclusion

In this work, we report new EXAFS results of PuO_2_ and CeO_2_ NPs with different average sizes and compare them with the published data for ThO_2_, and UO_2_ NPs. It was found that the changes observed in the spectra were the same for all series, emphasizing the similarities in the nature and behavior of these dioxide NPs. Only ThO_2_ demonstrates higher ordering in short-range oxygen surroundings, which is explained by its more amorphous nature, particularly with decreasing NP size. Plutonium demonstrated structural characteristics similar to those of the other studied MeO_2_ NPs.

A conceptual core-shell model with calculated effective CN_ef_ was proposed to fit the changes in CN for the Me–Me coordination sphere in the EXAFS spectra of MeO_2_ NPs. The proposed model perfectly correlates with the experimental results for all studied series and can be used in future studies of other substances.

## . Related literature

The following reference, not cited in the main body of the paper, has been cited in the supporting information: Langford *et al.* (1991 ▸).

## Supplementary Material

Sections S1 to S3; Table S1; Figures S1 to S6. DOI: 10.1107/S160057752101300X/yx5005sup1.pdf

## Figures and Tables

**Table 1:** List of the studied CeO_2_ and PuO_2_ samples

Sample	Synthesis procedure	Average particle size (nm)
CeO_2_-NPs-2.2 nm	From 0.05 *M* Ce(IV); dried at 40°C	2.2 ± 0.2
CeO_2_-NPs-2.5 nm	From 0.1 *M* Ce(IV); non-dried	2.5 ± 0.3
CeO_2_-NPs-2.9 nm	From 0.01 *M* Ce(III); dried at 40°C	2.9 ± 0.3
CeO_2_-NPs-5.7 nm	From 0.1 *M* Ce(III); dried at 40°C	5.7 ± 0.6
CeO_2_-NPs-6.3 nm	From 0.1 *M* Ce(IV); annealed at 400°C (12 h)	6.3 ± 0.8
CeO_2_-NPs-15 nm	From 0.8 *M* Ce(III); dried at 40°C	15 ± 2
CeO_2_-bulk	From 0.1 *M* Ce(IV); annealed at 1000°C (12 h)	>100
PuO_2_-NPs-2.0 nm	From 10^−4^ *M* Pu(VI), after 375 days at pH 12	2.0 ± 0.2
PuO_2_-NPs-2.2 nm†	From 6 × 10^−5^ *M* Pu(III) at pH 8†	2.2 ± 0.3
PuO_2_-NPs-3.2 nm	From 10^−4^ *M* Pu(VI), after 375 days at pH 8	3.2 ± 0.3
PuO_2_-bulk†	PuO_2_ reference (Oak Ridge National Laboratory, Batch ID No. Pu-242-327 A1)†	>100

† Data from Gerber *et al.* (2020 ▸).

**Table 2:** Structural parameters obtained from the fitting of EXAFS spectra

Sample	Coordination shell	Coordination number, CN	Interatomic distance, *R* (Å)	Debye−Waller factor (σ^2^) (Å^2^)	*R*-factor *k* range *R* range
CeO_2_-bulk	O	8†	2.34 ± 0.02	0.0097	0.044
	Ce	12†	3.84 ± 0.01	0.005	4–13
	O	24†	4.39 ± 0.08	0.014	1.5–4.2
CeO_2_-NPs-15 nm	O	8†	2.33 ± 0.02	0.009	0.032
	Ce	10.3 ± 0.8	3.84 ± 0.01	0.005†	4–13
	O	10.5 ± 15.1	4.43 ± 0.07	0.014†	1.5–4.2
CeO_2_-NPs-6.3 nm	O	8†	2.33 ± 0.01	0.0009	0.022
	Ce	8.4 ± 0.6	3.83 ± 0.01	0.005†	4–13
					1.5–4.2
CeO_2_-NPs-5.7 nm	O	8†	2.33 ± 0.02	0.0116	0.052
	Ce	8.0 ± 0.8	3.84 ± 0.01	0.005†	4–13
					1.5–4.2
CeO_2_-NPs-2.9 nm	O	8†	2.34 ± 0.02	0.0125	0.053
	Ce	5.1 ± 0.5	3.84 ± 0.01	0.005†	4–13
					1.5–4.2
CeO_2_-NPs-2.5 nm	O	8†	2.34 ± 0.02	0.0161	0.068
	Ce	3.8 ± 0.5	3.84 ± 0.01	0.005†	4–11
					1.5–4.0
CeO_2_-NPs-2.2 nm	O	8†	2.35 ± 0.02	0.0171	0.175
	Ce	2.6 ± 0.6	3.87 ± 0.02	0.005†	4–11
					1.5–4.0
PuO_2_-bulk	O	8†	2.33 ± 0.01	0.0061	0.016
	Pu	12†	3.82 ± 0.01	0.004†	3–14
	O	24†	4.39 ± 0.02	0.010	1.3–4.2
PuO_2_-NPs-3.2 nm	O	8†	2.32 ± 0.02	0.012	0.080
	Pu	4.7 ± 0.7	3.83 ± 0.01	0.004†	3–12
					1.3–4.0
PuO_2_-NPs-2.2 nm	O	8†	2.31 ± 0.01	0.011	0.028
	Pu	3.3 ± 0.4	3.81 ± 0.01	0.004†	3–12
					1.3–4.0
PuO_2_-NPs-2.0 nm	O	8†	2.31 ± 0.02	0.012	0.16
	Pu	1.7 ± 1.1	3.80 ± 0.03	0.004†	3–12
					1.3–4.0

† Parameters were fixed.

**Table 3:** Cell parameter and solubility product constant (*K*_sp_) for studied series CeO_2_–PuO_2_–ThO_2_–UO_2_

	Cell parameter, *A*	Reference	log*K*_sp_
PuO_2_	5.396	00-041-1170	−58.3 ± 0.5†
CeO_2_	5.4124	00-081-0792	−59.3 ± 0.3‡
UO_2_	5.466	00-078-0725	
ThO_2_	5.597	00-042-1462	−47.0 ± 0.8§

† Guillaumont *et al.* (2003 ▸).

‡ Plakhova *et al.* (2016 ▸).

§ Rand *et al.* (2008 ▸).
