# Supplementary material for: Effective coordination numbers from EXAFS: general approaches for lanthanide and actinide dioxides
Source: J Synchrotron Radiat. 2022 Jan 27;29(Pt 2):288–94. doi: 10.1107/S160057752101300X (PMC8900841; doi:10.1107/S160057752101300X)
Supplement: Supplementary file 1 [file s-29-00288-sup1.pdf]

JOURNAL OF SYNCHROTRON RADIATION

Volume 29 (2022)

Supporting information for article:

Effective coordination numbers from EXAFS: general approaches for lanthanide and actinide dioxides

Anna Romanchuk, Alexander Trigub, Tatiana Plakhova, Anastasiya Kuzenkova, Roman Svetogorov, Kristina Kvashnina and Stepan Kalmykov

S1. XRD data for CeO_2 and PuO_2 NPs

The experimental XRD patterns for CeO_2 and PuO_2 NPs are presented in Fig. S1.

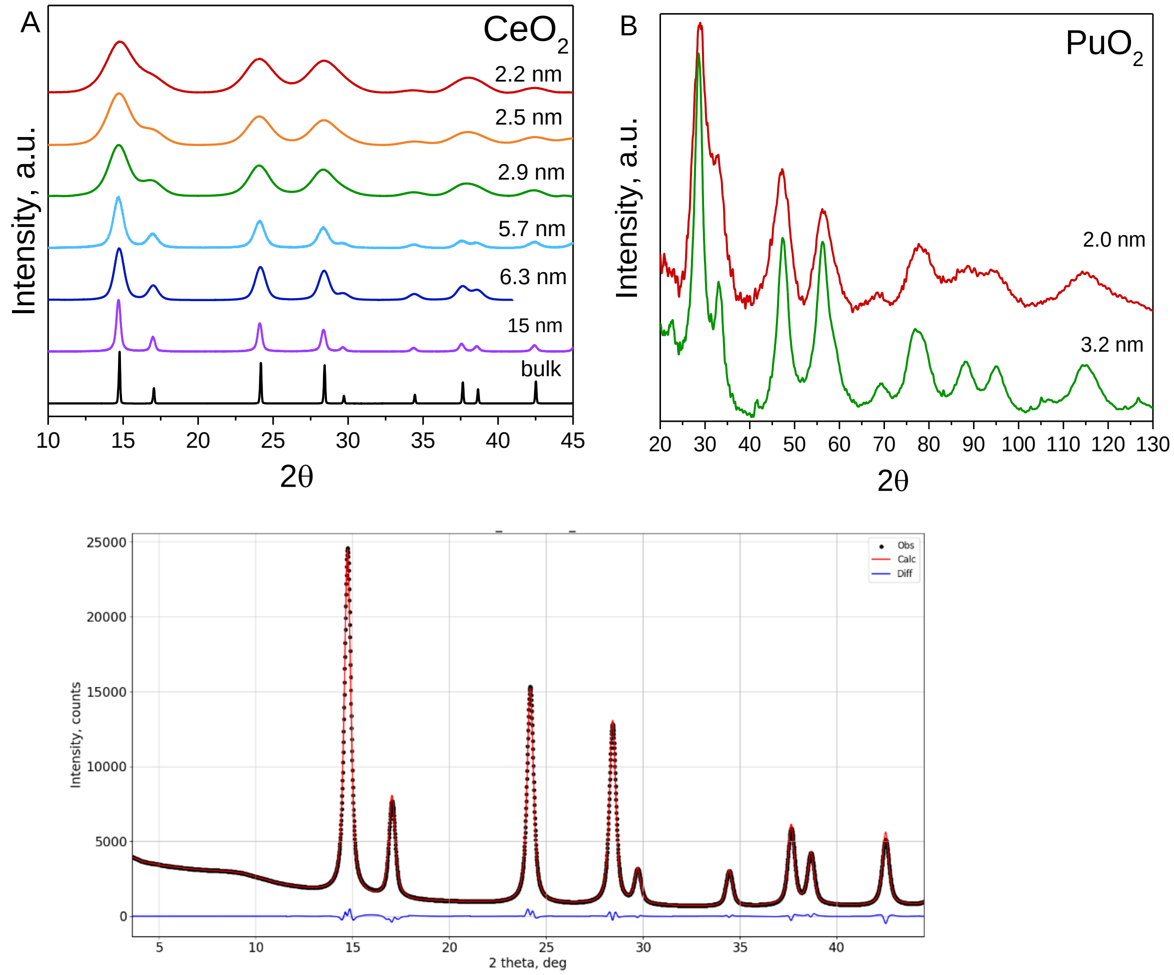

Figure S1 XRD data for studied (A) CeO_2 and (B) PuO_2 NPs. (C) Example of the results of Rietveld refinement CeO_2-15 nm sample.

Table S1 shows a comparison of CeO_2 NPs size determined by two different methods. The Scherrer equation can be represented as follows (1):

$$D = \frac{K\lambda}{\beta\cos(\theta)},$$

where D denotes crystallites size, λ is the wavelength (0.080 nm), K is the coefficient of anisotropy that is generally set as 0.94 for spherical crystals with cubic symmetry, θ is the scattering angle in radians, and β is the FWHM for the diffraction peak expressed in radians. Using the W–H plot, we

calculated the effective particle size considering the microstrains (ε) induced in crystallites owing to imperfections and distortions as follows:

$$\beta\cos\theta = \frac{K\lambda}{D} + 4\varepsilon\sin\theta$$

Table S1 Comparing of the size determination using different methods

Sample	Size Scherrer (error 10%)	Size W–H (error 10%)	Rietveld refinement
CeO_2-NPs-2.2 nm	2.2 ± 0.2	-	1.7
CeO_2-NPs-2.5 nm	2.5 ± 0.3	-	2.2
CeO_2-NPs-2.9 nm	2.9 ± 0.3	-	2.6
CeO_2-NPs-5.7 nm	5.7 ± 0.6	6.3 ± 0.7	6.0
CeO_2-NPs-6.3 nm	6.3 ± 0.8	7.3 ± 0.7	6.1
CeO_2-NPs-15 nm	15 ± 2	18 ± 2	17.8

As observed from the results, the sizes of the CeO_2 NPs calculated using different approaches are in good agreement. Notably, the W–H approach could not be applied to the ***CeO_2-NPs-2.2 nm, CeO_2-NPs-2.5 nm, and CeO_2-NPs-2.9 nm*** samples, as the slope in the W–H plot is negative in these cases. The negative slope indicates that the influence of microstrains is negligible, and the dominant source of broadening is the small crystallite size (Langford *et al.*, 1991). Thus, for the interpretation of the EXAFS results in the present work, the sizes calculated by the Scherrer formula were used, as they are more applicable in the case of small NP systems.

S2. XANES data

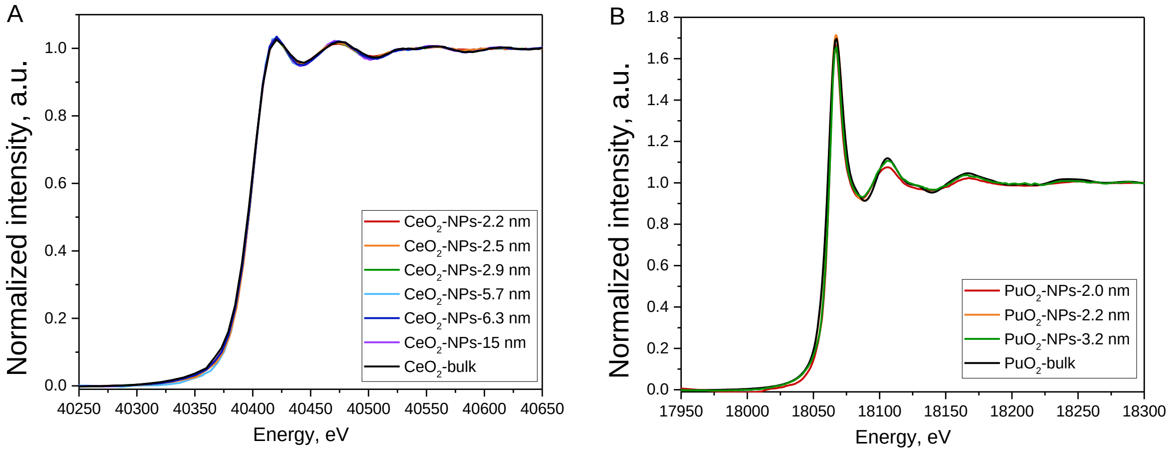

Figure S2 XANES spectra of studied (A) CeO_2 and (B) PuO_2 samples

S3. EXAFS fitting results

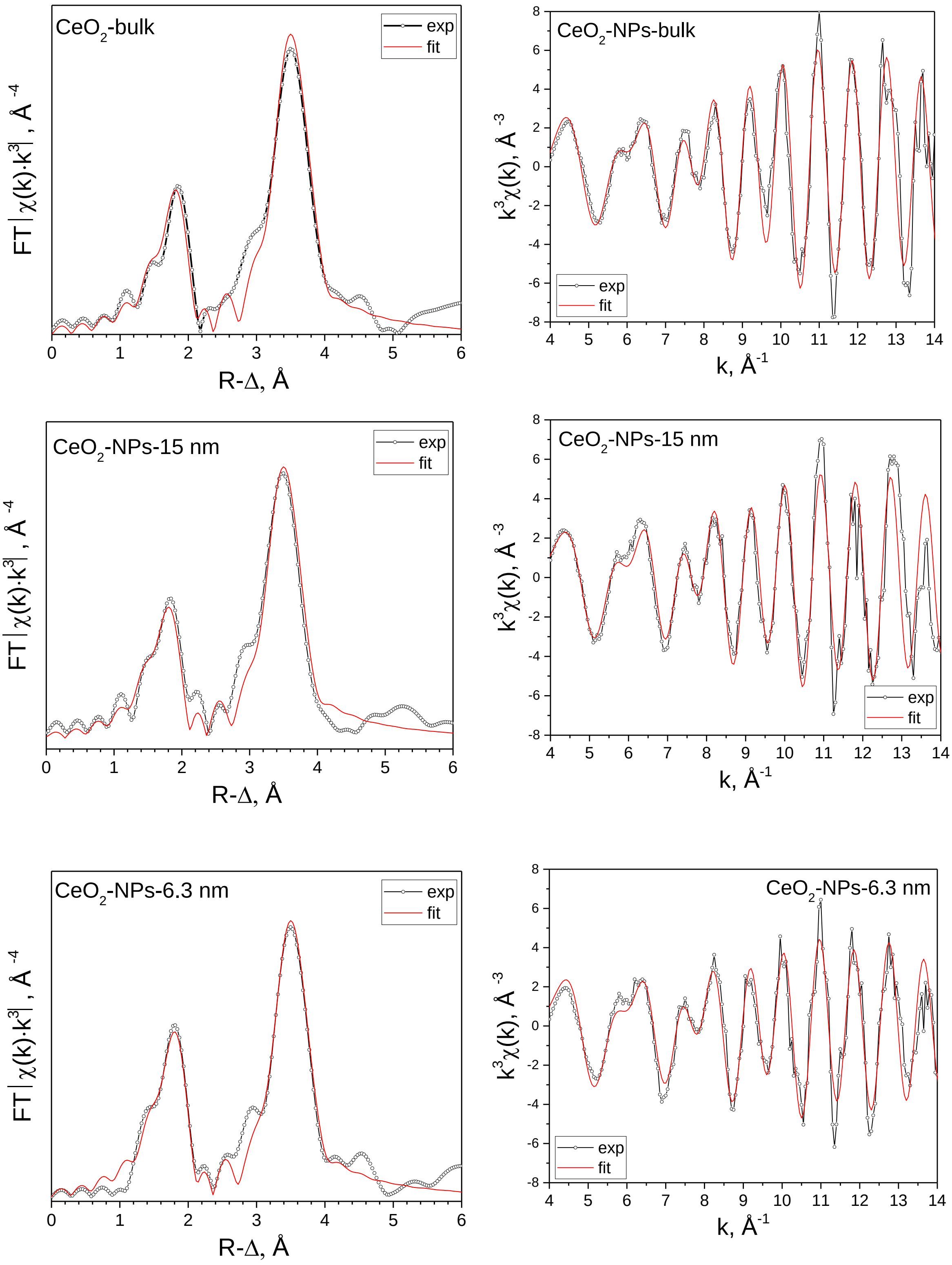

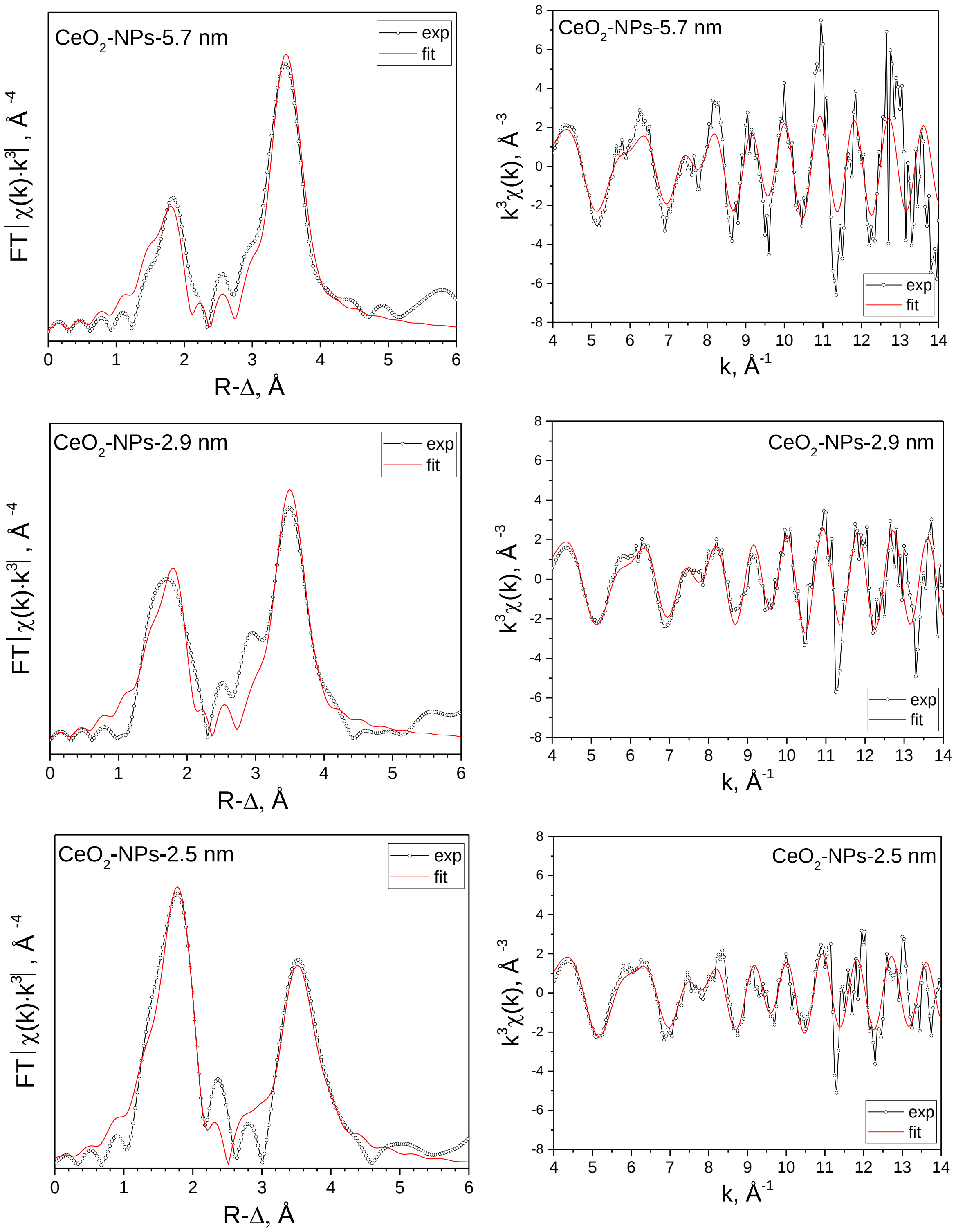

CeO2-NPs-5.7 nm
exp
fit
FT|χ(k)·k3|, Å-4
R-Δ, Å
CeO2-NPs-5.7 nm
exp
fit
k3χ(k), Å-3
k, Å-1
CeO2-NPs-2.9 nm
exp
fit
FT|χ(k)·k3|, Å-4
R-Δ, Å
CeO2-NPs-2.9 nm
exp
fit
k3χ(k), Å-3
k, Å-1
CeO2-NPs-2.5 nm
exp
fit
FT|χ(k)·k3|, Å-4
R-Δ, Å
CeO2-NPs-2.5 nm
exp
fit
k3χ(k), Å-3
k, Å-1

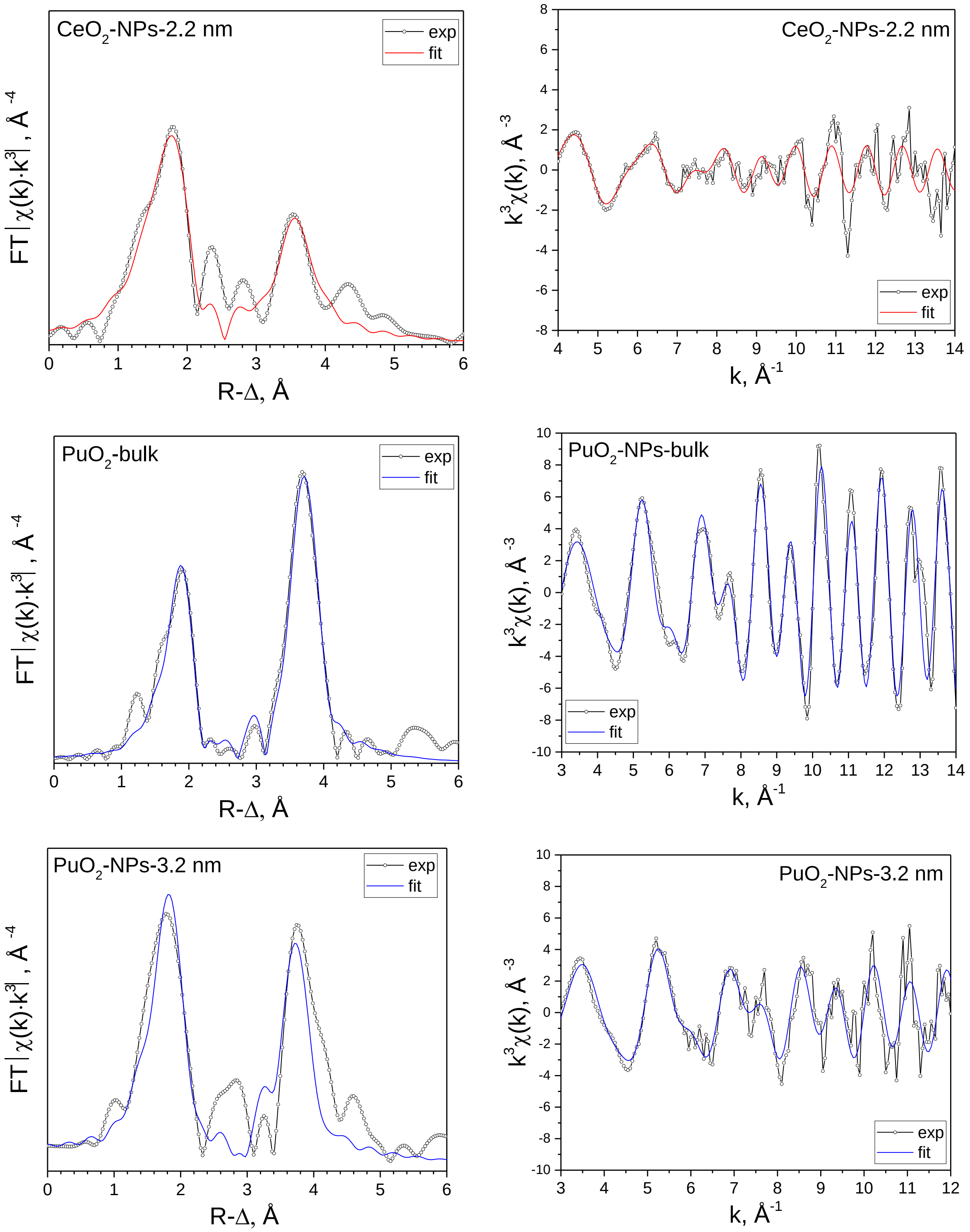
CeO2-NPs-2.2 nm
exp
fit
FT|χ(k)·k3| , Å -4
R-Δ, Å
CeO2-NPs-2.2 nm
k3χ(k), Å -3
k, Å-1
PuO2-bulk
exp
fit
FT|χ(k)·k3| , Å -4
R-Δ, Å
PuO2-NPs-bulk
k3χ(k), Å -3
k, Å-1
PuO2-NPs-3.2 nm
exp
fit
FT|χ(k)·k3| , Å -4
R-Δ, Å
PuO2-NPs-3.2 nm
k3χ(k), Å -3
k, Å-1

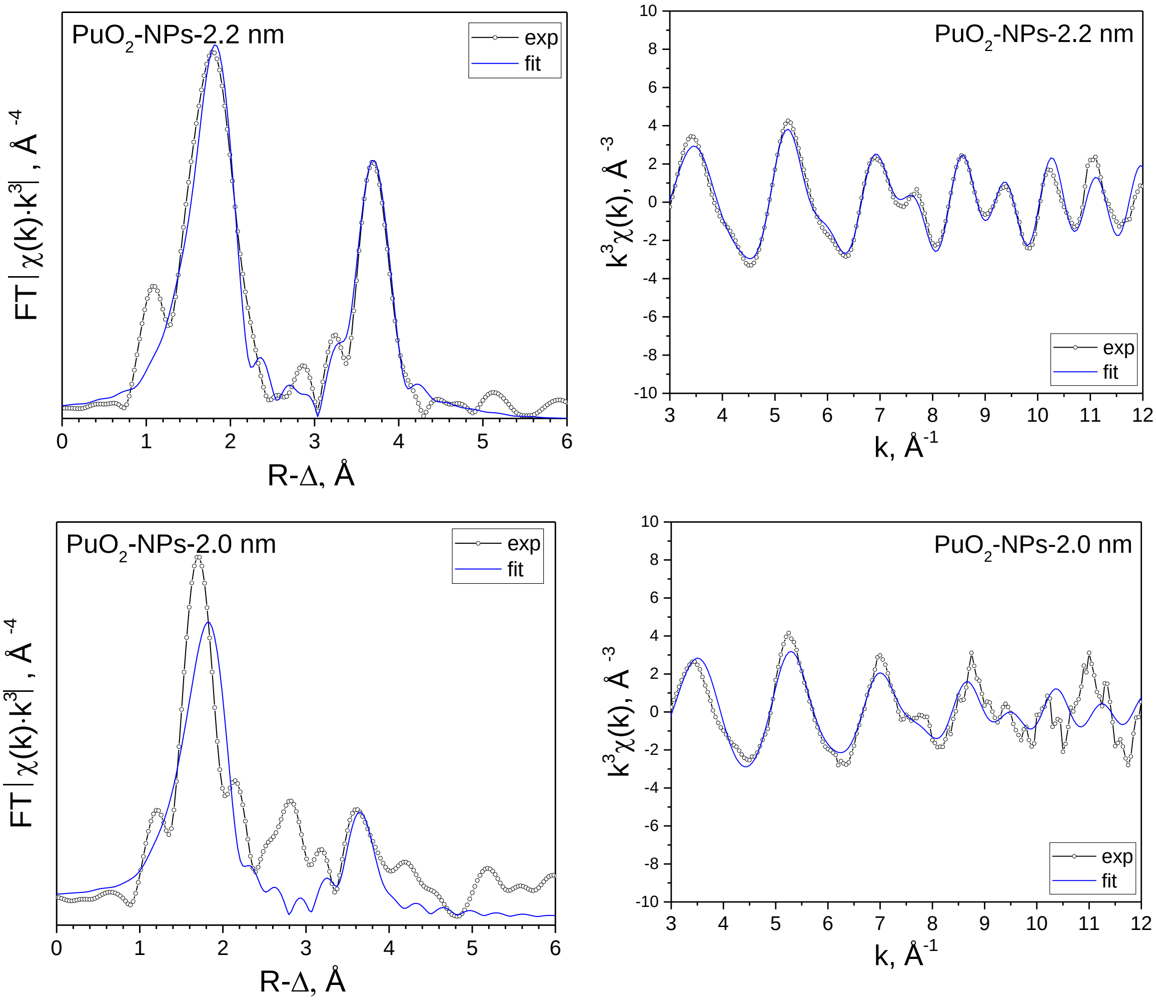

Figure S3 Result of fitting EXAFS spectra of CeO_2 and PuO_2 NPs

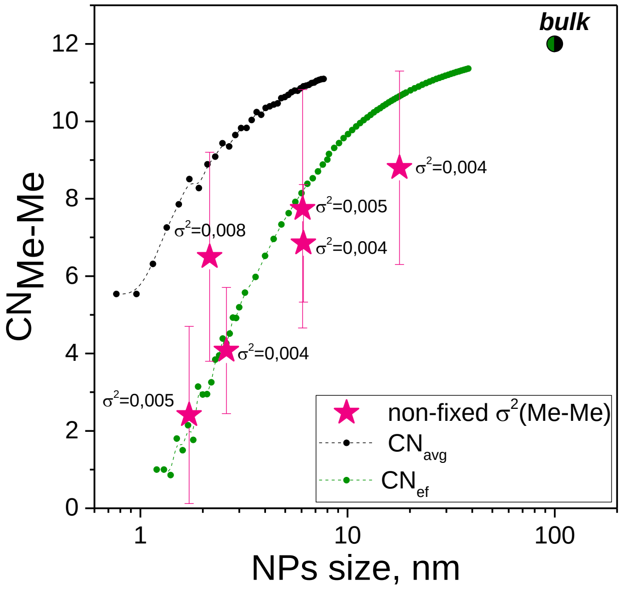

Figure S4 Comparison of the size-dependent change of Me–Me CN in CeO_2 NPs that were calculated without fixation of DW factor at 0.005.

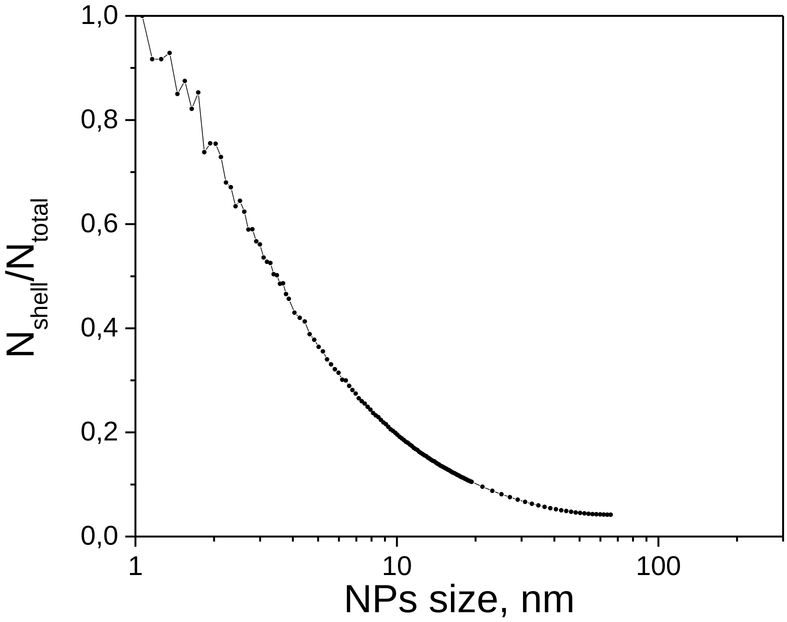

Figure S5 Dependence of relation shell atoms on the total number of atoms with increasing MeO_2 NP size.

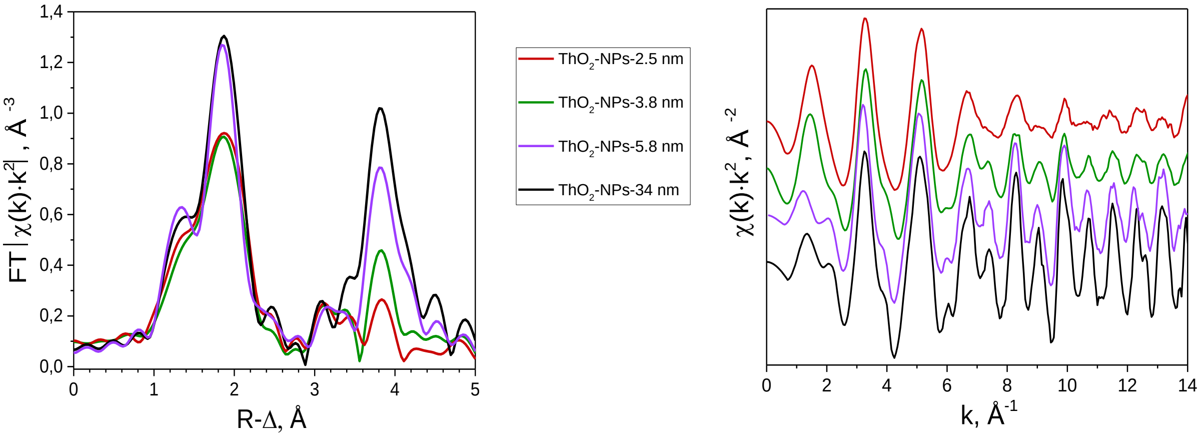

Figure S6 Th L_3-EXAFS: FT magnitude of EXAFS data (k – 3-13), and k^2-weighted $\chi(k)$ experimental functions from Plakhova *et al.*, 2019.
